# Does Family Medicine training in Thailand affect patient satisfaction with primary care doctors?

**DOI:** 10.1186/1471-2296-8-14

**Published:** 2007-03-29

**Authors:** Darin Jaturapatporn, Alan Dellow

**Affiliations:** 1 Family physician, Clinical lecturer, Department of Family Medicine, Ramathibodi Hospital, Mahidol University, Bangkok, Thailand; 2 General Practitioner, General Practice Trainer, Tutor for the Oxford Deanery, UK

## Abstract

**Background:**

Recent national healthcare reforms in Thailand aim to transfer primary care to family physicians, away from more expensive specialists. As Family Medicine has yet to be established as a separate discipline in Thailand, newly trained family physicians work alongside untrained general doctors in primary care. While it has been shown that Family Medicine training programs in Thailand can increase the quality of referrals from primary care doctors to specialists, information is lacking about whether such training affects the quality of patient care. In the Department of Family Medicine at Ramathibodi Hospital, trained family physicians work with residents and general doctors. Although this situation is not typical within Thailand, it offers us the opportunity to look for variations in the levels of satisfaction reported by patients treated by different types of primary care doctor.

**Methods:**

During a two-week period in December 2005, 2,600 questionnaires (GPAQ) were given to patients visiting the Department of Family Medicine at Ramathibodi Hospital. Patients were given the choice of whether or not they wanted to participate in the study. A cross-sectional analysis was performed on the completed questionnaires. Mean GPAQ scores were calculated for each dimension and scored out of 100. Student t-tests, ANOVA with F-test statistic and multiple comparisons by Scheffe were used to compare the perceived characteristics of the different groups of doctors. Five dimensions were measured ranging from access to care, continuity of care, communication skills, enablement (the patient's knowledge of a self-care plan after the consultation) and overall satisfaction.

**Results:**

The response rate was 70%. There were significant differences in mean GPAQ scores among faculty family physicians, residents and general doctors. For continuity of care, patients gave higher scores for faculty family physicians (67.87) compared to residents (64.57) and general doctors (62.51). For communication skills, patients gave the highest GPAQ scores to faculty family physicians (69.77) and family medicine residents (69.79). For enablement, faculty family physicians received the highest score (82.44) followed by family medicine residents (80.75) and general doctors (76.29).

**Conclusion:**

Faculty family physicians scored higher for continuity of care when compared with general doctors and residents. General doctors had lower GPAQ scores for communication skills and enablement when compared to faculty family physicians and residents. Faculty family physicians had the highest GPAQ scores in many dimensions of family practice skills, followed by residents and general doctors.

## Background

In the 1990s, in response to an economic crisis, the Government of Thailand reformed primary health care in an effort to reduce medical care costs [1]. Even though Family Medicine had never been established as a separate discipline in Thailand, the aim was to transfer most primary care to family physicians and away from more expensive specialists. One difficulty with this approach was the lack of family physicians, as as a consequence of the increasing specialization of Thailand's physicians. Since 1969, most graduate doctors have preferred to study in specialist areas such as Surgery, Internal Medicine, Orthopedics, and Obstetrics & Gynecology. Although three-year training programs in general practice were established in 1969, few doctors were trained in this area. In 1999, only 216 (1.7%) of the 12,500 Thai board-certified physicians were general practitioners [2]. Adding to the confusion, general doctors with no postgraduate training were also called general practitioners.

In August 1998, in the hope of attracting more doctors to primary care training, doctors completing training programs in Family Medicine were designated as Family Physicians to distinguish them from general doctors. In that year, nine residents joined the first year of the nationwide program. The number of residents joining these programs increased yearly, reaching a peak of 60 trainees in 2002. Despite this increase, family medicine is still not fully recognized as a discipline or specialty in Thai society. This leads us to question whether there are differences in the care provided by family physicians and general doctors working in the primary care setting and to ask whether Family Medicine training programs affect the quality of patient care. A recent Thai study showed that family medicine training programs can increase the quality of referrals from primary care doctors to specialists in terms of improved communication between both groups of doctors, fewer referrals and more positive assessments by specialists on the quality of referrals [3]. However, there is a lack of information in the literature about whether the quality of primary care is affected by family medicine residency training in Thailand. Our hypothesis is that such programs can increase patient satisfaction, an important component of quality in primary care.

This study was undertaken in the Department of Family Medicine at Ramathibodi Hospital in Bangkok, one of Thailand's leading medical schools. The Department of Family Medicine at Ramathibodi Hospital has provided family medicine residency training since 2001 and is one of the most popular family medicine programs in Thailand. With the exceptions of Paediatrics, Obstetrics, ENT, Dermatology and Ophthalmology cases, the out-patient unit of the Department of Family Medicine acts as a gatekeeper for almost all new patients attending the hospital. Fifty percent of patients are from the inner-city population, the remainder coming from across the country. Patients are seen randomly by faculty physicians, general doctors and family medicine residents working in the Department of Family Medicine. The definitions of each type of doctor are described as follows:

### Definitions of primary care doctors working in Thailand

**Faculty physicians**: doctors with a post-graduate qualification in Family Medicine/General Practice.

**General Doctors**: doctors with no post-graduate training in Family Medicine/General Practice.

**Residents**: doctors taking part in the 3-year postgraduate Family Medicine/General Practice training program.

The three groups of doctors generally practice medicine in a similar fashion, having the same authority to order tests, prescribe medication and make referrals to specialists.

Assessment of patient satisfaction is one way to determine the quality of primary care, although it is dependent on the service that patients utilize and the subjectivity of their opinions [4]. Patient satisfaction questionnaires can be used as a useful tool to evaluate the performance of medical students during consultations and also to measure patient satisfaction with different health care professional groups such as general practitioners, nurse practitioners, district nurses and practice nurses [5,6]. Comparisons between groups of health care professionals must be undertaken using well-recognized standardized tests. The General Practice Assessment Questionnaire (GPAQ) is a standard satisfaction questionnaire allowing patients to evaluate primary care in a number of key areas [7]. The questionnaire consists of seven multi-item scales ranging from the frequency of visits (item 1), the helpfulness of receptionists (item 2), access to healthcare (items 3–8), continuity of care (item 9), the doctor's communication skills (item 10), the patient's knowledge of a self-care plan after the consultation (item 11), overall satisfaction (item 12), demographic data (items 13–18) and general comments (item 19). All questions have 5–6 response scales and can be calculated as a GPAQ score, allowing services to be analysed and compared. The information obtained can then be used to make improvements. GPAQ was used to evaluate patient satisfaction in this study because of its quality and ready application for use. A Thai-version of GPAQ is a self-administered patient questionnaire translated from an English-version of GPAQ with the permission of the National Primary Care Research and Development Centre (NPCRDC) at the University of Manchester and following Guidelines for the process of Cross-Cultural Adaptation of Self-Report Measures [8-16]. The Thai version of GPAQ has been shown to achieve good levels of reliability and validity, with Cronbach's alpha coefficients being 0.8221[17].

## Methods

A cross-sectional analytic study was carried out using a Thai-version of GPAQ. Over a two week period in December 2005, GPAQ was given to patients attending the Department of Family Medicine at Ramathibodi Hospital upon completion of each consultation. Approximately 1,000 patients attended the primary care clinic each day. Every fourth consecutive patient was approached to join the study. Informed consent was received from 45 participating doctors before the study commenced, with no refusals to participate.

Patients agreeing to participate in the study were asked to put completed questionnaires into a collection box. Patients could refuse to participate at any time. This study included both follow-up and new patients. New patients were not permitted to choose which doctor they would see for the first time and were seen by the same doctor for subsequent visits. Only existing patients, therefore, could evaluate the doctor in terms of continuity of care.

To assess quality of care, five dimensions were measured ranging from access to care, continuity of care, communication skills, enablement (the patient's knowledge of a self-care plan after the consultation) and overall satisfaction. Mean GPAQ scores were calculated for each dimension. Finally, SPSS version 11.5 was used to determine Chi-squares, T-test values, ANOVA with F-test statistics, and eta squared as well as to perform multiple comparisons by Scheffe to compare the characteristics and quality of care among the different groups of doctors.

This research project was reviewed and approved by the research ethics committee at Ramathibodi Hospital at Mahidol University in 2005.

## Results

Of the 2,600 questionnaires that were distributed 1,820 were returned and analysed, a response rate of 70%. A total of 45 primary care doctors participated in this study, comprising 15 faculty family physicians, 10 general doctors and 20 residents. There were no statistically significant differences in the mean age of the faculty physicians, general doctors or residents (table 1). Faculty physicians, however, tended to be older, followed by general doctors and then residents. The majority of faculty physicians and general doctors were female, while there was an even mix of male and female residents.

**Table 1:** Characteristics of faculty physicians, general doctors and residents.

**Doctor's characteristics**	**Group**			**P-value**
		
	**Faculty physician (%) (N = 15)**	**General doctor (%) (N = 10)**	**Resident (%) (N = 20)**	
**Mean age (SD) years**	31.05 (10.57)	30.13 (9.46)	28.24 (2.24)	0.08
**Sex**				
Male	3	1	10	0.00
Female	12	9	10	
**Length of time in this workplace (SD) years**	2.71 (0.78)	2.30 (0.63)	2.05 (0.50)	0.10

The characteristics of patients examined by faculty physicians, general doctors and residents were also analyzed. There were no differences in terms of age, sex, payment status, presence or absence of chronic illness, address, type of accommodation, occupation or income (table 2). Residents took care of more new cases in comparison to the other groups of doctors. In Thailand, in addition to self-payment, there are 3 public risk protection schemes (medical care coverage) namely, a universal coverage scheme, a social security scheme and a civil servants' medical benefit scheme. 50% of patients visiting Ramathibodi Hospital were covered by the civil servants' medical benefit scheme, i.e. no limitation to the cost of treatment, while 35% paid for their own health care costs. Most patients (70%) were home owners. The majority of patients were female (71.03%) and housewives (31.30%). There were small numbers of unemployed and disabled patients in the study. 50% of patients coming to Ramathibodi Hospital had an average income of 10,000–30,000 Baht/month.

**Table 2:** Characteristics of patients seen by faculty physicians, general doctors and residents.

**Patient characteristics**	**Group**			**P-value**
		
	**Faculty physician (%)**	**General doctor (%)**	**Resident (%)**	
**Mean age (SD), years**	50.39 (15.65)	49.32 (15.86)	49.55 (15.39)	0.07
**Sex**				
Male	233 (30.50)	92 (24.90)	249 (31.50)	0.07
Female	530 (69.50)	277 (75.10)	542 (68.50)	
**Status of patients**				
New patients	113 (14.50)	75 (16.40)	235 (36.70)	0.00
Existing patients	665 (85.50)	382 (83.60)	405 (63.30)	
**Funding of patient**				
Universal coverage	37 (4.70)	8 (1.80)	30 (4.70)	0.10
Social welfare	62 (7.90)	38 (8.40)	38 (5.90)	
Government welfare	416 (53.10)	248 (54.70)	294 (45.80)	
Self-payment	269 (34.30)	159 (35.10)	280 (43.60)	
**Chronic illness**				
yes	259 (35.60)	141 (33.30)	199 (34.40)	0.68
no	469 (64.40)	286 (66.70)	379 (65.60)	
**Accommodation**				
Owner	566 (72.80)	322 (72.20)	436 (69.40)	0.36
Renting	212 (27.20)	124 (27.80)	192 (30.60)	
**Occupation**				
Business owner	51 (7.10)	33 (7.90)	40 (7.0)	0.47
Employee	155 (21.70)	98 (23.30)	154 (27.00)	
Government worker	121 (16.90)	81 (19.30)	95 (16.60)	
Student	39 (5.50)	20 (4.80)	33 (5.80)	
Housewife	234 (32.80)	134 (31.90)	167 (29.20)	
Unemployed	5 (0.70)	1 (0.20)	7 (1.20)	
Disabled	14 (2.00)	10 (2.40)	15 (2.60)	
Retired	95 (13.30)	43 (10.20)	60 (10.50)	
**Income (Baht/month)**				
< 5,000	50 (14.20)	68 (21.20)	33 (15.20)	0.13
5,001–10,000	113 (32.10)	106 (33.00)	66 (30.40)	
10,001–30,000	160 (45.50)	131 (40.80)	108 (49.80)	
30,001–50,000	21 (6.00)	11 (3.40)	6 (2.80)	
>50,000	8 (2.30)	5 (1.60)	4 (1.80)	

There were significant differences in mean GPAQ scores for the quality of care between the faculty physician, resident and general doctor groups (table 3) as assessed by ANOVA with F-test statistic and multiple comparisons by Scheffe and eta squared. By applying eta squared it can be seen that 15%, 17% and 7% respectively of the satisfaction scores for continuity of care, doctor's communication skill, and enablement can be explained by the different groups of primary care doctors.

**Table 3:** Comparison of general practice assessment scores for faculty physicians, general doctors and residents.

**Area of assessment**	**Group**			**F**	**P-value**	**Eta squared**
				
	**Faculty physician**	**General doctor**	**Resident**			
	**Score (SD)**	**Score (SD)**	**Score (SD)**			
**Access**	54.96 (13.20)	55.04 (11.89)	55.00 (12.94)	0.05	0.995	0.00
**Continuity of care**	67.87 (17.54)	62.51 (16.99)	64.57 (17.09)	10.316	0.000*	0.15
**Doctor's communication skill**	69.77 (14.24)	65.08 (14.20)	69.79 (14.25)	16.294	0.000*	0.17
**Enablement**	82.44 (24.56)	76.29 (28.10)	80.75 (25.84)	6.865	0.001*	0.07
**Overall satisfaction**	80.70 (14.37)	79.73 (14.10)	80.86 (15.50)	0.758	0.469	0.01

* statistical significance

For continuity of care, patients gave higher scores for faculty physicians (67.87) in comparison to residents (64.57) and general doctors (62.51). For communication skills, patients gave the highest GPAQ scores to faculty physicians (69.77) and residents (69.79). For enablement, both faculty physicians (82.44) and residents (80.75) received statistically significant higher scores in comparison to general doctors (76.29).

## Discussion

Patient satisfaction is a strong measure in the evaluation of quality of care because it reflects the experiences of those who receive the care [4]. Although positive feelings towards doctors can be affected by factors such as the personality of the doctor, the ability of the doctor to reassure, the nature of the patient's disease and characteristics of the patient, our data demonstrates that patients perceive differences between different types of primary care doctors in the primary care clinic.

In Thailand, every graduating doctor has to work for the government for three years before beginning a residency training program. In the case of family medicine, which is considered to be a shortage specialty, one can choose to train as a family physician without working for the government. Most of the younger generations of faculty family physicians have chosen this route. Therefore, the ages and years of experience of faculty physician were not significantly different from other groups of doctors in the study. The patients in this study appear relatively young, partly because the elderly tended to be unable to complete questionnaires by themselves. A previous study, however, also showed that patients visiting the same primary care clinic were relatively young with an approximate mean age of 50 years [3].

The strengths of this study are the good return rate (70%) and the quality of the Thai-version of GPAQ (Cronbach's alpha coefficient of 0.8221) [17]. One limitation is that Ramathibodi Hospital is a tertiary care hospital, so the result may not be generalisable to other primary care settings. Primary care doctors in Thailand usually work in clinics or community hospitals rather than in a tertiary care setting. Patients attending Ramathibodi Hospital tend to be more clinically complex, having consulted doctors at other clinics before coming to Ramathibodi Hospital. Also, 50% of patients visiting Ramathibodi Hospital were covered by the civil servants' medical benefit scheme, meaning that there is no limitation to the cost of treatment. In other primary care settings the majority of patients come under the universal coverage scheme, receiving limited standard care [18]. Even though 70% of patients were home owners, suggesting a relative wealthy group, 50% of patients had incomes between 10,000–30,000 Baht/month (£150–450), differing little from the £300/month average income for the Thai population [19].

In government hospital settings, such as Ramathibodi Hospital, patients usually have to wait for 1–3 hours before being seen by a doctor. Most patients anticipate having to wait for this period of time and tend to rate their satisfaction against this expectation. This might explain why there is no significant difference in overall satisfaction when viewing the rating scores on the access scale.

Patients are beginning to expect better communication skills from health care professionals [20]. This is an evolving process in Thailand, where doctors and nurses have traditionally been among the most respected members of society. Good doctor-patient communication makes Thai patients satisfied, while poor communication can lead to increasing litigation by patients against doctors [20]. There is a need, therefore, to improve doctors' communication skills at both the undergraduate and postgraduate education levels. This study demonstrates that Family Medicine residency training is associated with better reports of satisfaction from patients in primary care in terms of communication skills and patient enablement in comparison to general doctors who have not completed residency training programs. Residents were also evaluated higher than general doctors, although they had not finished the training program. This may reflect the characteristics of doctors who choose to undertaken this type of training, the selection criteria for such training programs or the educational impact of training itself. Although not statistical significant, there were some potential confounding factors in the study that have to be taken into account, such as the age and sex of the doctors, the length of time working in the Department of Family Medicine, and patient characteristics. Eta squared in this study shows the association of different types of primary care doctors with satisfaction scores in the range 0–17%. Additional factors, therefore, must also be taken into account. The aim of our study was notto discriminate between the quality of care of residents and general doctors but to demonstrate differences in quality of care that can be achieved by appropriate residency programs. These findings highlight the importance of Family Medicine residency training in producing a more effective primary care workforce in Thailand. Family Medicine is indeed a specialty, having specific requirements in terms of knowledge and skills, training needs, support and resources. We have shown that GPAQ scores can be used as a tool to evaluate and compare patient satisfaction as part of an assessment of the quality of care provided by different groups of primary care doctors.

## Conclusion

This study was conducted in a primary care clinic within a tertiary care hospital, an unusual setting for a primary care clinic in Thailand. In this setting, faculty physicians were scored higher for continuity of care when compared with general doctors and residents. General doctors had lower GPAQ scores for communication skills and enablement when compared with faculty doctors and residents. When assessed by patients, faculty physicians received higher GPAQ scores in many dimensions of family practice skills, followed by residents and then general doctors. For these results to be generalized, a study is needed on a more representative population of doctors.

## Competing interests

The author(s) declare that they have no competing interests.

## Authors' contributions

DJ participated in the design of the study and performed the statistical analysis. ACD participated in the tool development and helped to draft and edit the manuscript. Both authors read and approved the final manuscript.

## Pre-publication history

The pre-publication history for this paper can be accessed here:


